# Rejuvenating immunity: “anti-aging drug today” eight years later

**DOI:** 10.18632/oncotarget.3740

**Published:** 2015-03-31

**Authors:** Mikhail V. Blagosklonny

**Affiliations:** ^1^ Cell Stress Biology, Roswell Park Cancer Institute, Buffalo, NY, USA

**Keywords:** mTOR, TOR, gerosuppression, rapalogs, lifespan, longevity, diseases

## Abstract

The 2014 year ended with celebration: Everolimus, a rapamycin analog, was shown to improve immunity in old humans, heralding ‘a turning point’ in research and new era in human quest for immortality. Yet, this turning point was predicted a decade ago. But what will cause human death, when aging will be abolished?

“Defining ageing as a disease and then trying to cure it is unscientific and misguided.” [1]

## INTRODUCTION

Until recently, aging was believed to be a functional decline caused by accumulation of random molecular damage, which cannot be prevented.

Breaking this dogma, hyperfunction theory described aging as a continuation of growth, driven by signaling pathways such as TOR (Target of Rapamycin). TOR-centric model predicts that rapamycin (and other rapalogs) can be used in humans to treat aging and prevent diseases [2]. In proper doses and schedules, rapamycin and other rapalogs not only can but also must extend healthy life-span in humans [2, 3].

This theory was ridiculed by opponents and anonymous peer-reviewers. Yet, it was predicted in 2008 that “five years from now, current opponents will take the TOR-centric model for granted” [4]. And this prediction has been fulfilled.

## RAPAMYCIN TODAY

The study that Evirolimus (RAD001), a rapamycin analog, improves immunity in aging humans [5] made sensational headlines:

“Novartis Working on ‘Fountain of Youth’ Drug”. “Researchers could be closing in on a “fountain of youth” drug that can delay the effects of aging and improve the health of older adults”.

As summarized by Nir Barzilai, “it sets the stage for using this drug to target aging, to improve everything about aging. That's really going to be, for us, a turning point in research, and we are very excited.” http://www.medicaldaily.com/anti-aging-drug-works-first-steps-toward-boosting-immune-system-delaying-aging-315592

## RAPAMYCIN YESTERDAY

In 2006, it was concluded that “ Sirolimus or Rapamune, which is known in the basic science as rapamycin, is already approved for clinical use, available and can be used immediately. In addition to cancer, cardiovascular diseases, autoimmunity, and metabolic disorders, all diseases of aging from osteoporosis to Alzheimer's may be treated with rapamycin. Finally, rapamycin will be most useful as an anti-aging drug to slow down senescence and to prevent diseases.” [2]

And further, “Rapamycin is safe enough to be administrated daily to transplant patients for several years. Actually, rapamycin is so safe that its pharmacokinetics have been studied in healthy volunteers”. “Figuratively, it [rapamycin] transforms immunity from aged-type to infant-type”. In simple words, rapamycin rejuvenates the immunity. Thus, “rapamycin eliminates hyper-immunity rather than suppresses immunity” [2].

“Anti-aging drug today” [3] was actually published *yesterday:* “Rapamycin is a non-toxic, well-tolerated drug that is suitable for everyday oral administration. Preclinical and clinical data indicate that rapamycin is a promising drug for age-related diseases and seems to have anti-tumor, bone-sparing and calorie-restriction-mimicking ‘side-effects’.” [3]. As recently reviewed, in proper doses, lifespan-extending agents including rapamycin posses certain immunostimulatory activities [6].

By 2010, many predictions of the TOR-centric model have been tested and confirmed [7]. In 2010, one prediction remained: “rapamycin will become the cornerstone of anti-aging therapy in our life time.” [7]. Until December 2014, all gerontological papers on rapamycin stated that current rapalogs are just proof of principle and will not be used due to side effects. Even further, use of anti-aging drugs in our lifetime was called science fiction. For unclear reasons, scientists emphasized that rapamacin and other current rapalogs will not be used in aging humans due to imaginary side effects.

## TRIUMPH OF MTOR-CENTRIC MODEL

The hyperfunctional theory predicts calorie-restriction-mimicking ‘side-effects’ of rapalogs. For example, rapamycin increases lipolysis, thus imitating fasting [3]. And in some conditions, rapamycin may cause “starvation diabetes”, a benevolent insulin-resistance and glucose intolerance. “Starvation or Hunger Diabetes” was well known during famine and prolonged fasting [8]. Rapamycin, as calorie-restriction-mimetic, can cause starvation-like symptoms in certain conditions. This benevolent rapamycin-induced state prevents complications of true type II diabetes [9, 10]. In certain strains of mice, rapamycin causes some symptoms of starvation-like insulin-resistance, erroneously viewed as real diabetes [11]. These metabolic alterations are reversible [12, 13]. MTOR-centric model predicts that this reversible insulin resistance is benevolent and is associated with increased longevity because longevity is promoted not via increased insulin sensitivity, but instead via decreased mTOR pathway signaling [9].

Initially, mTOR-centic model was ignored. As announced by Lamming et al, “A growing list of side effects make it doubtful that rapamycin would ultimately be beneficial in humans.” [14] Now however the same opponent re-invented mTOR-centric model (without appropriate reference), suggesting that “longevity is promoted not via increased insulin sensitivity, but instead via decreased PI3K/Akt/mTOR pathway signaling” [15]. As it was predicted in 2008 [4], opponents indeed take mTOR-centric model for granted. This is the ultimate triumph of the TOR-centric (hyperfunction) theory of aging.

## TOR-CENTRIC MODEL

Evolutionary theory predicts that growth-promoting pathways are antagonistically pleiotropic [16]. In other words, growth-promoting signaling is essential during development and may be harmful later in life. In particular, the nutrient-sensing mTOR pathway is essential for growth and development. In adults, its excessive activity leads to pathology (aging) [17-20]. Aging is an unintended, harmful continuation of developmental growth. It is a quasi-program (not a program), a shadow of development. More on that was discussed previously [16, 21-27].

## THREE SOURCES FOR THE TOR-CENTRIC MODEL

### 1. Genetics of longevity

The work in model organisms revealed numerous genes whose inactivation extends life span [3, 28-57]. Some gerogenes encode the TOR pathway. Yet, is the TOR pathway central or just one of the numerous pathways? Independent work on cellular senescence answers this question.

### 2. Cellular senescence

In 2003 it was proposed that activation of growth-promoting pathways should cause senescence, when the cell cycle is blocked [58]. In fact, mTOR converts reversible cell cycle arrest to cellular senescence (geroconversion) [59-61]. Rapamycin partially suppresses geroconversion [62-76]. All gerogenic pathways converge on the mTOR pathway: upstream and downstream [77-83]. Typically, oncogenes are gerogenes, whereas tumor suppressors are gerosuppressors [59, 84-87]. Gerogenes and gerosuppressors constitute the mTOR network. This network is identical to gerogenic pathways identified in model organisms [3].

### 3. Diseases

Completely independently, mTOR pathway was revealed in the studies of human diseases: Parkinson and Alzheimer, cancer and benign tumors, cardiac fibrosis and atherosclerosis, renal hypertrophy and diabetic complications [19, 88-97]. And while gerontologists thought that rapamycin causes cancer, numerous studies by nephrologists and transplantologists showed that rapamycin prevents cancer in humans [86, 98-103]. Most studies were performed by scientists, working in narrow clinical fields [104-106]. Only taken together, these studies illuminate the role of mTOR in all age-related diseases. These age-related diseases are direct causes of death in aging. No one dies from aging per se.

Michael Hall, who discovered TOR and named it after rapamycin [107], remarkably envisioned in 2005 that “inhibitors of mammalian TOR may be useful in the treatment of cancer, cardiovascular disease, autoimmunity, and metabolic disorders” [93]. This generalization, combined with discoveries in model organisms and cellular senescence, was taken a step further [2]. mTOR-driven hyperfubnction leads to alterations of homeostasis, diseases and death. Examples of systemic hyperfuctions include hypertension, hyper-insulinemia and organ hypertrophy.

## HOW WILL PEOPLE DIE, WHEN AGING WILL BE ABOLISHED: POST-AGING SYNDROME

Currently, humans and animals (in protected environment) die from age-related diseases, which are manifestation of aging. By slowing aging, rapamycin and calorie restriction can delay age-related diseases including cancer [108-125]. They extend life span. Yet, the causes of death seem to be the same. Or not? Why is this important?

Consider an analogy. 300 years ago in London, 75% of people died from external causes (infections, trauma, starvation) before they reached the age of 26. [22]. So only a few died from mTOR-driven aging. Only when most external causes have been eliminated, people now die from mTOR-driven age-related diseases. Similarly, if TOR-driven aging would be eliminated by a rational combination of anti-aging drugs, even then we still would not be immortal. There will be new, currently unknown causes of death. I call this post-aging syndrome. We do not know what it is. But we know that accumulation of molecular damage or telomere shortening (as examples) eventually would cause post-aging syndrome [2].

## WHY DO WE NOT RECOGNIZE SYMPTOMS OF POST-AGING SYNDROME?

Even in the ancient world, when most people died from “external causes”, symptoms of mTOR-driven aging were well known. In contrast, we do not know symptoms of post-aging syndrome. Aging is quasi-programmed and is not accidental. Although its rate varies among individuals, the chances to outlive aging and to die from post-aging syndrome are very low. Still, we may identify these symptoms in humans over 110 years old and especially in animals treated with rapamycin (and other anti-aging modalities). Inhibition of mTOR may extend life span, thus revealing post-aging syndrome.

How will we know that we observe post-aging syndrome? There are potential criteria: Animals and humans die from either unknown diseases, unusual variants of known-disease and rare diseases. Or at least, the range of age-related diseases is dramatically changed.

As discussed in 2006, causes of post-aging syndrome may include accumulation of random molecular damage, telomere shortening, selfish mitochondria and so on. As also discussed, when people die from post-aging syndrome, then anti-oxidants may help, in theory of course [2].

## CONCLUSION

Criticizing the TOR-centric hyperfunction theory of aging, an opponent wrote: “The advent of the hyperfunction theory of aging has been compared to the replacement of the geocentric with the heliocentric worldview. Within this rather grand conceptual framework, I may be seen as an old-timer who desperately tries to salvage a doomed theory by piling up epicycles. Perhaps so – time will tell.” [126].

Time told unexpectedly fast. While gerontologists were studying free radicals and anti-oxidants, the TOR-centric (hyperfunction) theory revealed anti-aging drugs such as rapamycin and metformin. There are several potential anti-aging drugs in clinical use [127, 128]. Combining drugs and modalities, selecting doses and schedules in clinical trial will ensure the maximal lifespan extension [27, 129].

Simultaneously, medical progress improves aging-tolerance [129, 130]. Aging tolerance is the ability to survive despite aging [130]. For example, bypass surgery allows patients with coronary disease to live, despite aging-associated atherosclerosis.

Gerontologists do not need to catch the train that has already departed. No need to study rapamycin, which already entered the clinic. This is now a merely medical task. Gerontologists may continue to study free radicals and accumulation of random molecular damage as a potential cause of post-aging syndrome (not aging). It is important to study post-aging syndrome, to be ready to fight it, when medical progress with rapamycin will allow us to reach post-aging age: perhaps 50 years from now.
